# Inhibition of Cathepsin S Produces Neuroprotective Effects after Traumatic Brain Injury in Mice

**DOI:** 10.1155/2013/187873

**Published:** 2013-10-24

**Authors:** Jianguo Xu, Handong Wang, Ke Ding, Xinyu Lu, Tao Li, Jiawei Wang, Chunxi Wang, Jian Wang

**Affiliations:** ^1^Department of Neurosurgery, Jinling Hospital, School of Medicine, Nanjing University, 305 East Zhongshan Road, Nanjing, Jiangsu 210002, China; ^2^Department of Anesthesiology and Critical Care Medicine, Johns Hopkins University, School of Medicine, Baltimore, MD 21218, USA

## Abstract

Cathepsin S (CatS) is a cysteine protease normally present in lysosomes. It has long been regarded as an enzyme that is primarily involved in general protein degradation. More recently, mounting evidence has shown that it is involved in Alzheimer disease, seizures, age-related inflammatory processes, and neuropathic pain. In this study, we investigated the time course of CatS protein and mRNA expression and the cellular distribution of CatS in a mouse model of traumatic brain injury (TBI). To clarify the roles of CatS in TBI, we injected the mice intraventricularly with LHVS, a nonbrain penetrant, irreversible CatS inhibitor, and examined the effect on inflammation and neurobehavioral function. We found that expression of CatS was increased as early as 1 h after TBI at both protein and mRNA levels. The increased expression was detected in microglia and neurons. Inhibition of CatS significantly reduced the level of TBI-induced inflammatory factors in brain tissue and alleviated brain edema. Additionally, administration of LHVS led to a decrease in neuronal degeneration and improved neurobehavioral function. These results imply that CatS is involved in the secondary injury after TBI and provide a new perspective for preventing secondary injury after TBI.

## 1. Introduction

Cathepsin S (CatS) belongs to the family of lysosomal cysteine proteases that are normally present in lysosomes. It is initially synthesized in the rough endoplasmic reticulum as pre-proCatS and translocated via the Golgi by the mannose-6-phosphate pathway for storage in lysosomal compartments as an inactive zymogen [1]. CatS becomes active after removal of the propeptide by other proteases and autocatalytic cleavage facilitated by low lysosomal pH in the lysosome compartments [2].

Under physiological conditions, CatS is a potent protease that degrades intracellular proteins engulfed by lysosomes and extracellular elements such as elastin, fibronectin, laminin, and collagens [3]. Apart from its role in protein degradation, it is crucial to the immune response because it degrades the invariant chain II, an essential step in major histocompatibility complex (MHC) class II antigen presentation [4]. The latest research has shown that the activity of CatS is regulated by gamma-interferon-inducible lysosomal thiol reductase in B cells [5].

Studies have also shown that CatS is involved in a number of different pathologic processes. Požgan et al. [6] found that CatS is significantly elevated in synovial fluid of patients with rheumatoid arthritis, and Williams et al. [7] reported that CatS plays a central role in ozone-induced airway hyperresponsiveness and inflammation. CatS has also been implicated in metabolic disturbances such as obesity [8], diabetes [9], and dyslipidemia [10]. Several lines of evidence suggest that CatS is causally involved in the development of cardiovascular disease through stimulation of atherosclerotic plaques, destabilization of advanced plaques [11], and participation of left ventricular remodeling after myocardial infarction [12]. Moreover, CatS activity has also been proposed to play a part in the development of cancer by promoting cancer cell migration and tumor angiogenesis [13]. Mounting evidence also indicates that CatS has a complex relationship with autophagy in cancer and other diseases [14–16].

In the nervous system, increased CatS expression has been associated with Alzheimer disease [17], kainate-induced seizures [18], and age-related inflammatory processes [19]. It is also thought to contribute to neuropathic pain [20, 21]. Thus CatS appears to play important roles in inflammation, particularly in chronic pathologic processes. However, data are sparse regarding the interaction between CatS and acute inflammation, such as that which occurs after traumatic brain injury (TBI). In this study, we investigated how TBI affects the expression of CatS protein and mRNA in the cortex of mice and the cellular distributions of CatS after TBI. In addition, we used the nonbrain penetrant, irreversible CatS inhibitor morpholinurea-leucine-homophenylalanine-vinylsulfone-phenyl (LHVS) to clarify the relationship between CatS and secondary injury after TBI.

## 2. Materials and Methods

### 2.1. Animals

Male ICR mice weighing 28–32 g (Experimental Animal Centre of Nanjing Medical University, Jiangsu, China) were used in this study. Experimental protocols were approved by the Animal Care and Use Committee of Nanjing University and conformed to the Guide for the Care and Use of Laboratory Animals published by the National Institutes of Health. The mice were housed on a 12 h light/dark cycle with free access to food and water.

### 2.2. Model of TBI

We used Marmarou's weight-drop model with some modifications previously described by Flierl et al. [22]. Mice were anesthetized with an intraperitoneal injection of chloral hydrate (1%, 5 mL/kg) and then placed onto the platform directly under the weight of the weight-drop device. A 1.5 cm midline longitudinal scalp incision was made and the skull exposed. After locating the left anterior frontal area (1.5 mm lateral to the midline on the midcoronal plane) as the impact area, a 200 g weight was released and dropped onto the skull from a height of 2.5 cm. Mortality rate from apnea was reduced by early respiratory support. The scalp wound was closed with standard suture material, and the mice were returned to cages, where they had free access to water and food. Sham-injured animals underwent the same procedures but did not undergo the weight drop.

### 2.3. LHVS Administration

A subset of mice received intracerebroventricular injections of CatS inhibitor LHVS (PolyPeptide Group, Strasbourg, France) or vehicle (20% Cremophor EL/saline; Sigma, St. Louis, MO, USA) 10 min before TBI. A Hamilton syringe was used to deliver 10, 30, or 50 nM LHVS in 6 *μ*L of vehicle to the right (contralateral) lateral ventricle at the following stereotactic coordinates: 0.1 mm posterior and 1.0 mm lateral of the bregma, 3.0 mm in depth. The control group received 6 *μ*L of vehicle in the same way. Delivery of LHVS or vehicle was performed over 5 min, and the syringe was left in place for an additional 10 min to prevent reflux. Dosages were based on prior investigations in the collagen-induced arthritis model and our preliminary study [21].

### 2.4. Brain Tissue Preparation

For isolation of CatS protein and mRNA, animals were deeply anesthetized with a solution of chloral hydrate at 1, 3, 6, 12, or 24 h after TBI and perfused intracardially with 30–40 mL of cold (4°C) heparinized 0.9% saline. The left (ipsilateral) cerebral cortex was collected, immediately frozen in liquid nitrogen, and then transferred to a −80°C freezer until use. For immunohistochemistry, animals were sacrificed 3 h after TBI. After being deeply anesthetized with chloral hydrate, animals were perfused intracardially with 30–40 mL of cold heparinized 0.9% saline followed by 20–30 mL of cold 4% paraformaldehyde. The whole brain was removed and immersed in 4% paraformaldehyde overnight. For double immunofluorescence, the brain was subsequently immersed in 20% sucrose followed by 30% sucrose. For Fluoro-Jade C (FJC) staining, animals were sacrificed at 24 h after TBI, and tissue was collected as for immunofluorescence.

### 2.5. Western Blot Analysis

Protein concentrations were determined by the Bradford method [23]. Equal amounts of protein (100 *µ*g) per lane were separated by 10% sodium dodecyl sulfate-polyacrylamide gel electrophoresis and transferred to polyvinylidene-difluoride (PVDF) membranes. The membranes were blocked for 2 h in blocking buffer (Tris-buffered saline/0.05% Tween 20 (TBST) containing 5% skim milk) and then incubated overnight at 4°C with primary antibodies against CatS (1 : 500; Santa Cruz Biotechnology, Santa Cruz, CA, USA) and *β*-actin (1 : 5000; Bioworld Technology, Minneapolis, MN, USA) in blocking buffer. After being washed with TBST (3 × 10 min), the membranes were incubated with goat anti-rabbit horseradish peroxidase-conjugated IgG (1 : 5000; Bioworld Technology) for 2 h at room temperature. The protein bands were visualized by enhanced chemiluminescence (ECL) Western blot detection reagents (Millipore, Billerica, MA, USA) and exposure to X-ray film. Developed films were digitized on an Epson Perfection 2480 scanner (Seiko Corp., Nagano, Japan). Band density was quantified by using Un-Scan-It 6.1 software (Silk Scientific Inc., Orem, UT, USA); data were normalized to *β*-actin and expressed as percentage of sham.

### 2.6. Real-Time Quantitative Polymerase Chain Reaction (PCR)

Total RNA was extracted from ipsilateral cortex samples with RNAiso Plus (TaKaRa Bio, Dalian, China). The concentration and purity of total RNA were determined with a spectrophotometer (OD260/280 1.8–2.0) and 1% agarose gel electrophoresis. To avoid RNA degradation, some of the RNA was immediately reverse transcribed to cDNA with the PrimeScript RT reagent kit (TaKaRa Bio, Dalian, China), and the surplus RNA was kept at −80°C. The primers were designed according to PubMed GenBank and synthesized by Invitrogen Life Technologies (Shanghai, China). The primer sequences were as follows: CatS: F: 5′-CCATTGGGATCTCTGGAAGAAAA-3′; R: 5′-TCATGCCCACTTGGTAGGTAT-3′; *β*-actin: F: 5′-AGTGTGACGTTGACATCCGTA-3′; R: 5′-GCCAGAGCAGTAATCTCCTTCT-3′. The quantitative real-time PCR analysis was performed by using the Mx3000P System (Stratagene, San Diego, CA, USA), applying real-time SYBR Green PCR technology. The PCR amplification program consisted of an initial denaturation step of 95°C for 30 s, followed by 40 cycles of 95°C for 5 s, and a 30 s annealing and elongation step at 60°C. All samples were analyzed in triplicate. *β*-actin was used as an endogenous reference “housekeeping” gene. Relative change in CatS mRNA expression after TBI was determined by the equation: fold change = 2^−[ΔΔCt]^, where ΔΔCt = (CtCatS − Ct*β*-actin)TBI − (CtCatS − Ct*β*-actin)Sham. Ct value is the cycle number at which fluorescence signal crosses the threshold.

### 2.7. Immunohistochemistry and Double Immunofluorescence

Consecutive 4 *µ*m thick serial sections were cut and routinely deparaffinized. Endogenous peroxidase was blocked with 3% H_2_O_2_/methanol. Nonspecific antibody binding was blocked by incubating the sections in blocking buffer (10% normal goat serum in phosphate-buffered saline (PBS)) for 30 min. Primary antibodies against CatS (1 : 100; Santa Cruz Biotechnology) were applied overnight at 4°C. After being washed three times in PBS for 5 min each, the sections were incubated with horseradish peroxidase-conjugated IgG (1 : 500; Santa Cruz Biotechnology) for 60 min. 3,3-Diaminobenzidine (DAB)/H_2_O_2_ solution was used to visualize CatS. Before the sections were mounted, cell nuclei were counterstained with hematoxylin.

For immunofluorescence, 8 *μ*m thick cryostat frozen sections were mounted on gelatin-coated slides, which were warmed at room temperature for 30 min. Slides were washed three times in PBS for 10 min each before immunofluorescence staining. Based on the established immunostaining protocol [24], slides were incubated in blocking buffer (10% normal goat serum in PBS containing 0.1% Triton X-100) for 2 h followed by overnight incubation at 4°C in primary antibodies to CatS (1 : 100; Santa Cruz Biotechnology), ED1 (1 : 100; Millipore), NeuN (1 : 100; Millipore), and glial fibrillary acidic protein (GFAP; 1 : 200; CST, Danvers, MA, USA). The next day, the slides were washed with PBS three times for 5 min and then incubated with appropriate secondary antibodies (goat anti-rabbit IgG-FITC, 1 : 200; Santa Cruz or Alexa Fluor 594, 1 : 200; Invitrogen, Grand Island, NY, USA) for 1 h at room temperature. The slides were washed three times in PBS, then counterstained with DAPI for 2 min, and rinsed with PBS. Cover slips were applied with mounting media. Fluorescence was imaged on an Olympus IX71 inverted microscope system and analyzed by Image-Pro Plus 6.0 software (Media Cybernetics, USA). The specificity of the immunofluorescence reaction was evaluated by replacement of the primary antibody with PBS.

### 2.8. Enzyme-Linked Immunosorbent Assay (ELISA)

Interleukin (IL-)1*β* and tumor necrosis factor (TNF-)*α* levels were analyzed by ELISA (Biocalvin Company, Suzhou, China) according to the manufacturer's instructions. Protein concentration was measured by using the Bradford method. Equal amounts of lysate were used for TNF-*α* and IL-1*β* analysis. Values are expressed as pg/mg protein.

### 2.9. Brain Water Content

Twenty-four hours after TBI, mouse brains were removed and placed on a cooled brain matrix. The brain stem and cerebellum were removed, and the left cerebral hemispheres were separated. The wet weight (ww) of each hemisphere was measured. Then the hemispheres were dried at 80°C for 72 h, and dry weight (dw) was measured. We calculated water content as a percentage using the following formula:

(1) $$\begin{array}{c} \frac{\text{ww}-\text{dw}}{\text{ww}}*100; \end{array}$$

see [25].

### 2.10. Fluoro-Jade C Staining and Cell Counting

Frozen sections were prepared, fixed, and mounted on slides as for immunofluorescence. FJC staining was performed according to a standard protocol [23, 26]. In brief, slides were first immersed in a basic alcohol solution consisting of 1% sodium hydroxide in 80% ethanol for 5 min. They were then rinsed for 2 min each in 70% ethanol and distilled water and then incubated in 0.06% potassium permanganate solution for 10 min. Following a 1-2 min water rinse, the slides were transferred for 10 min to a 0.0001% solution of FJC (Chemicon, Temecula, CA, USA) dissolved in 0.1% acetic acid vehicle. The slides were then rinsed through three changes of distilled water for 1 min per change. The slides were air-dried, cover slips were applied, and sections were visualized on an Olympus IX71 inverted microscope system. A pathologist blinded to the experimental group counted FJC-positive cells in the section through the injury's epicenter. The extent of brain damage was evaluated by the total number of FJC-positive cells in six fields (at 200x magnification) within the pericontusional cortex.

### 2.11. Neurobehavioral Evaluation

The neurologic status of the mice was evaluated at 24 h and 3 days after TBI by using the neurological severity score (NSS) and grip test. With the NSS, the investigator evaluates the ability of each mouse to perform 10 different tasks that demonstrate motor function, balance, and alertness. One point is given for failing to perform each of the tasks; thus, 0 = minimum deficit, and 10 = maximum deficit (Table 1) [22]. The severity of injury is defined by the initial NSS, evaluated 1 h after TBI, and is a reliable predictor of the late outcome. The grip test was developed on the basis of the test of gross vestibulomotor function [27]. The mouse is placed on a thin, horizontal, metal wire (45 cm long) that is suspended between two vertical poles 45 cm above a foam pad. The mouse is graded on its ability to grip, attach, and move as described in Table 2. The grip test was performed in triplicate, and a total value was calculated for each mouse. All neurobehavioral tests were carried out by an investigator who was blinded to the experimental groups.

### 2.12. Statistics

Each experiment was repeated at least three times, and the data are reported as the mean ± SEM. For the behavior tests, we used two-way ANOVA followed by Bonferroni post hoc test; for other assays, we used one-way ANOVA followed by Tukey's test. SPSS 17.0 (SPSS Inc., Chicago, IL, USA) was used for the statistical analysis. A *P* value < 0.05 was considered statistically significant.

## 3. Results

### 3.1. Expression of CatS Protein after TBI

**Figure 1:**
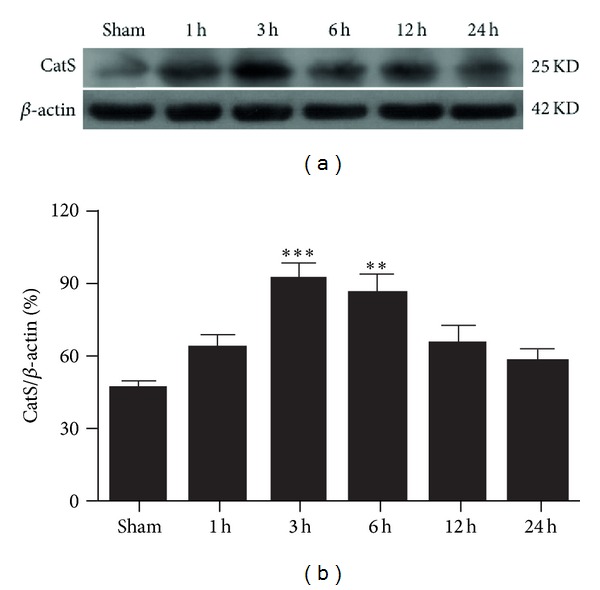
Time course of cathepsin S (CatS) protein expression in the ipsilateral cortex of mice after traumatic brain injury (TBI). Expression levels of CatS were normalized to those of *β*-actin. The level of CatS protein was significantly increased at 3 h and 6 h after TBI. (a) A typical Western blot result. (b) Quantification of data in (a). Data represent mean ± SEM (*n* = 6). **P < 0.01, ***P < 0.001 versus sham group.

CatS protein was expressed at a low level in the sham-TBI group but was elevated at 1 h after TBI, with a peak at 3 h (Figure 1). CatS protein expression was significantly higher in the TBI group than in the sham group at 3 h (*P* < 0.001) and 6 h (*P* < 0.01) and then began to decrease.

### 3.2. Expression of CatS mRNA after TBI

**Figure 2:**
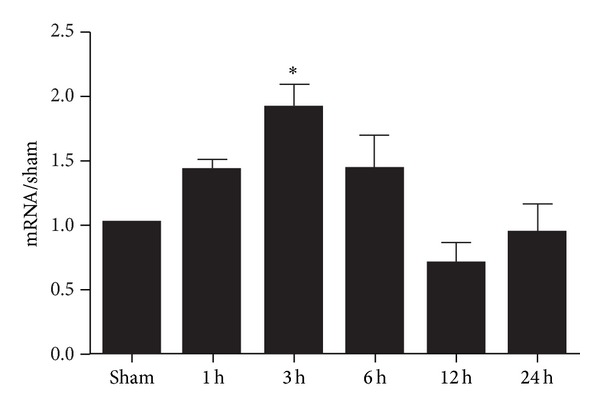
Time course of cathepsin S (CatS) mRNA expression in the ipsilateral cortex of mice after traumatic brain injury (TBI). The level of CatS mRNA was significantly increased at 3 h after TBI as compared with that in the sham group. *β*-actin was used as an internal control. The data were normalized to *β*-actin expression and were expressed as fold increase of the sham group. Data represent mean ± SEM (*n* = 6). *P < 0.05 versus sham group.

The changes in CatS protein level after TBI may result from activation of pre-CatS. Therefore, we used real-time quantitative PCR to determine whether CatS expression also changed at the mRNA level. CatS mRNA began to increase as early as 1 h after TBI (Figure 2). At 3 h after TBI, it reached a peak that was approximately 2-fold the level of the sham group (*P* < 0.05). The CatS mRNA level had begun to decrease by 6 h, and by 12 h, it was not different from that of the sham group (*P* > 0.05).

### 3.3. Immunohistochemistry of CatS after TBI

**Figure 3:**
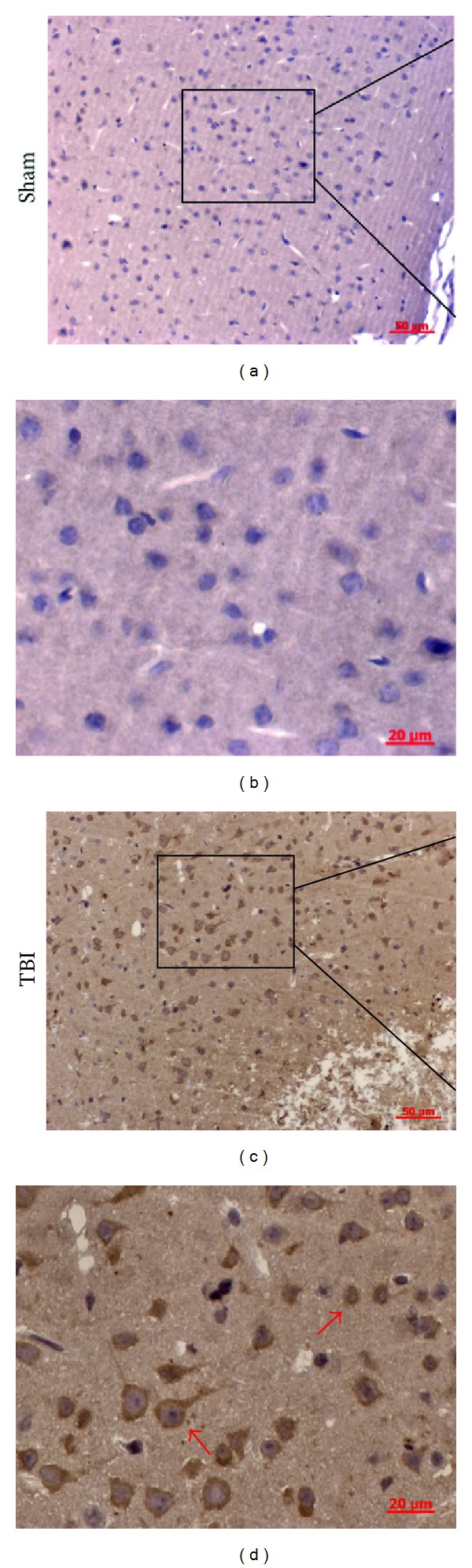
Representative photomicrographs showing cathepsin S (CatS) immunohistochemistry of tissue from the sham and traumatic brain injury (TBI) groups. In the sham group, almost none of the cells presented a positive morphology, whereas, in the TBI group, many cells were positive for CatS. CatS was present mainly in the cytoplasm. The CatS-positive cells in the TBI group (indicated by arrows) showed two populations of cells with different morphologies.

We used immunohistochemistry to further clarify the change in expression of CatS protein. As shown in Figure 3, CatS was weakly expressed in the cortex of the normal (sham) mouse brain. At 3 h after TBI, the expression of CatS was significantly increased in the pericontusional cortex, mainly in the cytoplasm. The CatS-positive cells in the TBI group (indicated by arrows) showed two populations of cells with different morphologies, which might represent microglia and neurons, respectively. When the primary antibody was omitted from the immunostaining reaction, no immunoreactivity was present, suggesting high specificity of the antibody to the antigen (data not shown).

### 3.4. Double Immunofluorescence for CatS and Neural Cell Markers

**Figure 4:**
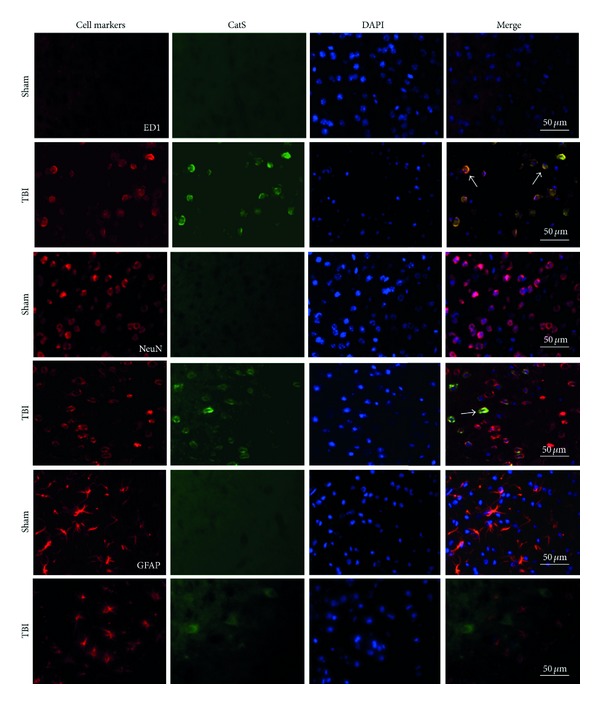
Representative photomicrographs of brain tissue from mice at 3 h after traumatic brain injury (TBI) or sham injury. Images show double immunofluorescent staining for cathepsin S (CatS; green) and different neural cell markers (red). Nuclei were counterstained with DAPI in the same view in each section. CatS staining was weak in the sham group but enhanced in the cytoplasm of the TBI group. Arrows indicate colocalization of CatS and ED1 or NeuN. Scale bars: 50 *µ*m.

To clarify the neural expression of CatS after TBI, we performed double immunofluorescent staining for CatS and cell-specific markers ED1, NeuN, and GFAP. As shown in Figure 4, CatS was expressed weakly in the sham group, consistent with the results of immunohistochemistry. However, CatS-positive cells were present in tissue from the 3 h after TBI group. The morphology indicated that CatS was expressed in cytoplasm. Overlapping images show that CatS was mainly expressed in ED1-positive cells and to a lesser extent in NeuN-positive cells. These results suggested that CatS is expressed mainly in microglia in the cortex 3 h after TBI.

### 3.5. LHVS Attenuates Production of Proinflammatory Cytokines in the Injured Brain Tissue

**Figure 5:**
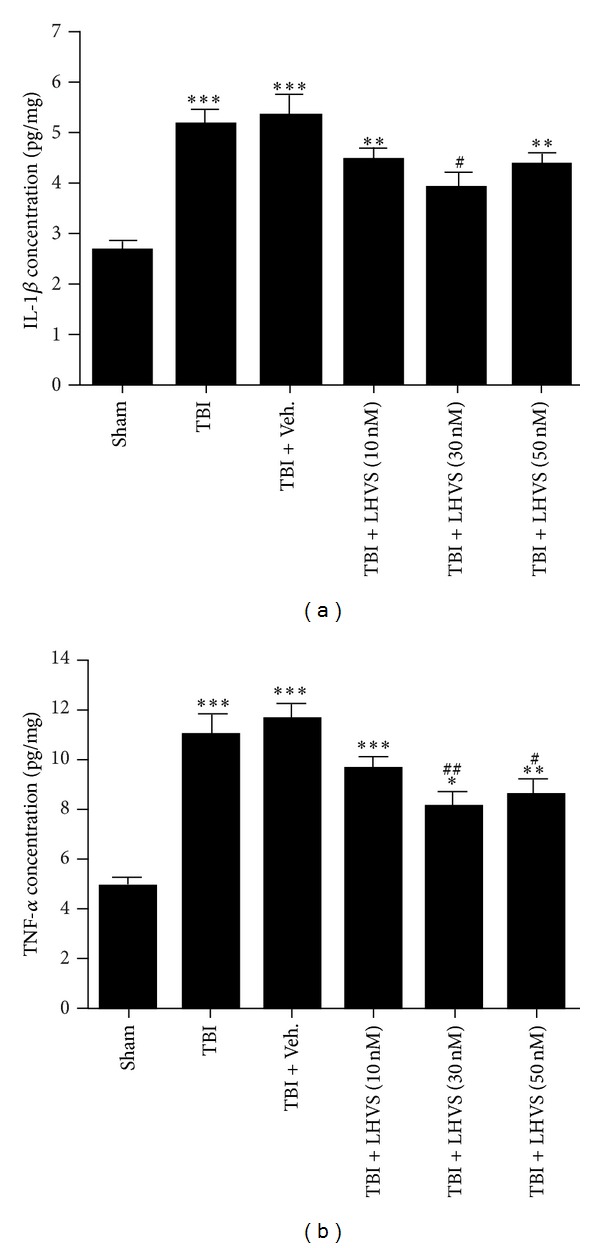
LHVS suppresses the upregulation of proinflammatory cytokines (IL-1*β* and TNF-*α*) in the ipsilateral cortex at 24 h after traumatic brain injury (TBI). (a) IL-1*β* concentration was increased significantly in the TBI and TBI + vehicle groups compared with that in the sham group. Pretreatment with LHVS significantly attenuated IL-1*β* levels in the ipsilateral cortex compared with vehicle pretreatment. (b) TNF-*α* concentration was increased significantly in the TBI and TBI + vehicle groups compared with that in the sham group. Pretreatment with LHVS significantly attenuated TNF-*α* levels in the ipsilateral cortex compared with vehicle pretreatment. Data are presented as mean ± SEM (*n* = 6). ***P < 0.001, **P < 0.01 versus sham group; ^#^ *P* < 0.05, ^##^ *P* < 0.01 versus TBI + vehicle group.

To evaluate the relationship between CatS and inflammation, we used six groups of mice (*n* = 6/group) as follows: sham group, TBI group, TBI + vehicle, and three TBI + LHVS groups (10, 30, and 50 nM). We used ELISA to detect the levels of proinflammatory factors at 24 h after TBI. IL-1*β* and TNF-*α* each increased significantly after TBI. No difference was observed between the vehicle-treated group and the TBI group (Figures 5(a) and 5(b)). Concentrations of IL-1*β* and TNF-*α* were reduced in the LHVS-treated groups compared to concentrations in the vehicle-treated group. Treatment with 30 nM LHVS produced a significant decrease in IL-1*β* (*P* < 0.05 versus TBI + vehicle; Figure 5(a)), and treatment with 30 nM and 50 nM LHVS produced significant decreases in TNF-*α* (*P* < 0.01 and *P* < 0.05, resp., versus TBI + vehicle; Figure 5(b)).

### 3.6. Pretreatment with LHVS Alleviates Brain Edema after TBI

**Figure 6:**
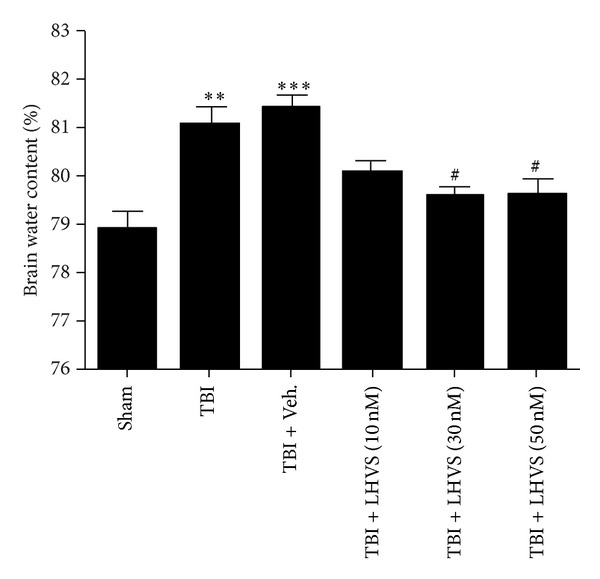
LHVS reduces cerebral edema in the ipsilateral cortex 24 h after traumatic brain injury (TBI). Brain water content was significantly higher in the TBI and TBI + vehicle groups than in the sham group. Pretreatment with LHVS significantly attenuated brain water content in the ipsilateral cortex compared with vehicle pretreatment. Data are presented as mean ± SEM. **P < 0.01, ***P < 0.001 versus sham group (*n* = 5); ^#^ *P* < 0.05 versus TBI + vehicle group.

To confirm the protective effect of LHVS at the macroscopic level, we measured the brain water content of mice pretreated with vehicle or LHVS. Six groups were used as described in Section 3.5 (*n* = 5/group). Compared with the sham group, mice in the TBI and TBI + vehicle groups had significantly greater brain water content. The brain water content did not differ significantly between the TBI group and the TBI + vehicle group. However, consistent with the changes observed in proinflammatory factors, groups treated with 30 nM and 50 nM LHVS had significantly less brain water content than did the TBI + vehicle group (*P* < 0.05; Figure 6).

### 3.7. LHVS Administration Suppresses Neuronal Degeneration in the Injured Brain

**Figure 7:**
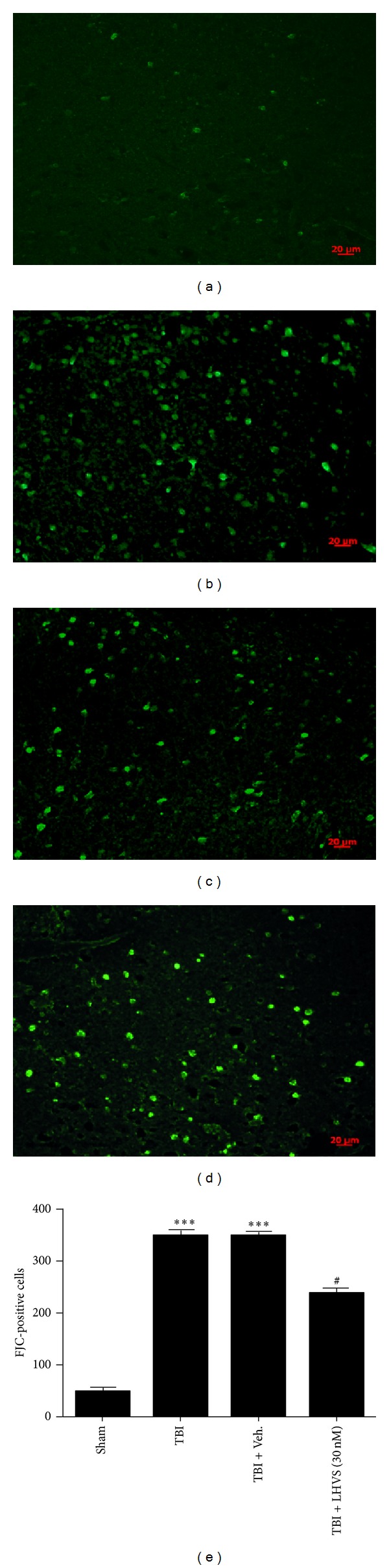
LHVS pretreatment reduces neuronal degeneration after traumatic brain injury (TBI). Upper panel: Fluoro-Jade C (FJC) staining of the pericontusional cortex in the sham group (a), TBI group (b), TBI + vehicle group (c), and TBI + LHVS (30 nM) group (d). Bottom panel: quantification of neurodegenerating (FJC-positive) cells in each group (*n* = 6 mice per group). Pretreatment with 30 nM LHVS significantly reduced the number of degenerating neurons at 24 h after TBI. Vehicle treatment had no effect. Data are presented as mean ± SEM (*n* = 6). ***P < 0.001 versus sham group; ^#^ *P* < 0.05 versus TBI + vehicle group. Scale bar: 20 *µ*m.

Next we investigated the role of CatS in neuronal degeneration after TBI by using FJC staining to determine how LHVS pretreatment affects neuronal degeneration in the cortex at 24 h after TBI. We chose 30 nM LHVS because the results described above showed it to be the most effective concentration. Few FJC-positive neurons were observed in the cortex of sham-TBI mice (Figure 7(a)). TBI caused a significant increase in the number of degenerating neurons in the cortex (*P* < 0.001 versus sham group; Figure 7(b)). Vehicle treatment had no effect on the number of degenerating neurons (*P* > 0.05 versus TBI group; Figure 7(c)). However, LHVS treatment significantly reduced the number of degenerating cortical neurons compared with that in the vehicle-treated group (*P* < 0.05; Figure 7(d)). These results indicate that administration of LHVS can reduce neuronal degeneration in the cortex surrounding the contusive lesion and provide neuroprotective effects after TBI.

### 3.8. LHVS Improves Neurobehavioral Performance of Mice after TBI

**Figure 8:**
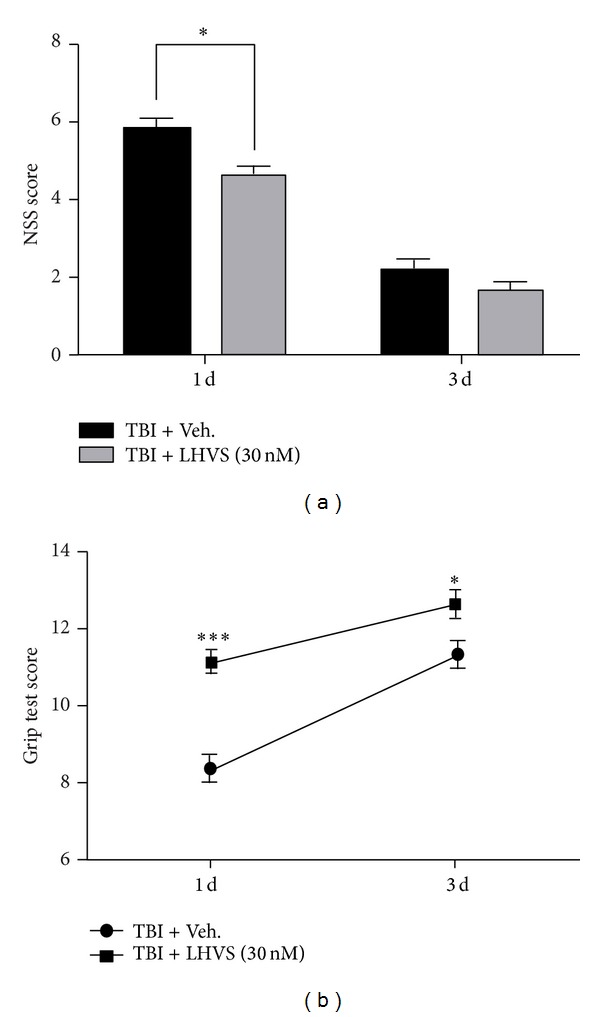
Neurological severity score (NSS) and grip test. (a) At 24 h after traumatic brain injury (TBI), mice pretreated with LHVS had significantly better neurological function than did mice pretreated with vehicle, as assessed by the NSS (a) and the grip test (b). At 3 days after TBI, function had improved in both groups. At this time point, neurological function was still slightly better in the LHVS-pretreated group, but the difference was significant only in the grip test. Data are presented as mean ± SEM (*n* = 6). *P < 0.05, ***P < 0.001 versus TBI + vehicle; two-way ANOVA followed by Bonferroni post hoc test.

At 24 h after TBI, the NSS score of the LHVS-treated mice was significantly better than that of the vehicle-treated mice (Figure 8(a)). At 3 days, the scores of both groups had improved, and although a difference was still detectable, it was not statistically significant. In the grip test, the scores of the LHVS-treated mice were significantly better than those of the vehicle-treated mice at both 24 h and 3 days after TBI (Figure 8(b)). As in the NSS, the scores of both groups were better at 3 days than at 24 h.

## 4. Discussion

In the present study, we investigated the influence of TBI on the expression of CatS in the ipsilateral cortex and the neuroprotective effect of administering the nonbrain penetrant, irreversible CatS inhibitor LHVS 10 min before TBI. The main findings of this study are as follows. (1) Expression levels of CatS protein and mRNA increase as early as 1 h after TBI and reach a peak at 3 h. (2) Consistent with prior studies [17, 19], CatS is expressed primarily in microglia cytoplasm and to a lesser extent in neuronal cytoplasm. (3) Administration of LHVS 10 min before the onset of TBI produces neuroprotection; specifically, it attenuates the production of inflammatory factors, alleviates cerebral edema, suppresses neuronal degeneration, and improves neurobehavioral performance.

It is a growing consensus that oxidative stress is a key component of the secondary injury cascade that follows TBI [28]. The brain is very sensitive to oxidative damage because it has a high content of polyunsaturated fatty acids, which are vulnerable to free radical attacks and lipid peroxidation [29]. A significant increase in oxidative damage can be observed as early as 30 min after trauma [30]. Oxidative stress after TBI induces peroxidation of cellular and vascular structures, protein oxidation, DNA cleavage, inhibition of the mitochondrial electron transport chain [28], and lysosome destabilization [31]. As a result of hyperpermeability, CatS may leak from lysosomes into the cytosol or extracellular space [31]. Though an endogenous inhibitor of CatS exists, it cannot inhibit excess CatS under pathological conditions [32].

Our data showed that the protein expression of CatS in the brain cortex upregulates as early as 1 h after injury and reaches a peak at 3 h. We speculated that the increase was related to the activation and release of the resident pool of CatS caused by oxidative stress. Two mechanisms may be involved in the process of CatS mRNA upregulation. First, a reduction in the CatS resident pool can increase mRNA expression. Second, ample evidence indicates that TBI can cause an increase in the expression of transcriptional factors such as AP-1 and NF-kB in a matter of minutes [33–36]. The products of these transcriptional factors may modify the regulation of other genes, and it is possible that CatS mRNA expression early after TBI is under such transcriptional control.

CatS is believed to be selectively expressed by antigen-presenting cells (APCs), including B-lymphocytes, macrophages, and microglia [37, 38]. In these cells, CatS is involved in adaptive immune responses that contribute to the process of antigen presentation, such as formation of specific peptides from foreign proteins [39] and degradation of the MHC II-associated chaperone invariant chain (li) from MHC II [2, 40]. However, apart from its roles in APCs, CatS has also been found in cerebral neurons of patients with neurodegenerative diseases and in the brains of aging mice [17, 19], where it is thought to be related to chronic inflammatory processes. 

Along with oxidative stress and glutamate excitotoxicity, inflammation is believed to be a key process in the secondary injury of TBI [41]. The inflammatory response that begins within minutes of traumatic impact to the brain [42, 43] can continue for hours or weeks. This inflammation causes neuronal degeneration and cytotoxicity directly and secondarily produces damage such as blood-brain barrier leakage that aggravates brain edema [44]. Substantial evidence has shown that suppressing the inflammation induced by TBI improves neurobehavioral outcome [45, 46].

Previous studies have shown that CatS may be involved in inflammation. Williams et al. [7] showed that inhibition of CatS downregulated proinflammatory cytokines in ozone-induced airway inflammation. Cattaruzza et al. [47] showed that CatS is active during colitis and exacerbates inflammatory processes by a mechanism dependent on protease-activated receptor 2. Therefore, we examined whether inhibition of CatS can reduce the level of IL-1*β* and TNF-*α* after TBI. Our results show that a dose of 30 nM LHVS can significantly downregulate proinflammatory cytokines, reduce cerebral edema, reduce neuronal degeneration, and improve neurologic function. These findings indicate that inhibition of CatS provides a neuroprotective effect that might result from suppression of the inflammatory response.

The CatS inhibitor LHVS is a synthetic dipeptide that has been demonstrated to selectively inhibit CatS [48]. Its inhibitory effect is due to the vinyl sulfone moiety attached to the carboxyl group of LHVS [49]. LHVS cannot penetrate the blood-brain barrier [20], and it may inhibit CatS by inhibiting its release from intracellular pools and inhibiting extracellular enzymatic activity [50]. 

Our study has a number of potential limitations. We chose to deliver LHVS directly into the lateral cerebral ventricle because it cannot infiltrate the blood-brain barrier, and we administered it 10 minutes before the onset of TBI to prevent LHVS reflux from trauma-induced intracranial hypertension. This protocol limits its application in clinical practice. Second, the peak effect of CatS was at 3 h after TBI, which would provide only a short window of opportunity for therapeutic interventions. Moreover, LHVS showed a beneficial effect over a relatively narrow dose range. Therefore, studies are needed to confirm that LHVS has a definite neuroprotective effect; in addition, multiple administration of LHVS will be considered to improve efficacy in our studies. Convincing evidence indicates that CatS is associated with chronic pathological processes, such as chronic pain and kainate-induced seizures [18, 20]. Chronic pain and seizures are both prevalent in patients recovering from TBI [51, 52]. Interestingly, Barclay et al. [20] showed that CatS expression progressively increased from day 3 to day 14 after injury. Whether CatS contributes to the delayed brain injury after TBI needs further study.

## 5. Conclusion

Taken together, our results show that CatS becomes upregulated at a subcellular level in the acute stage after TBI. Enhanced CatS expression was observed in the cytoplasm of microglia and to a lesser extent in neurons. Inhibition of CatS alleviated cerebral edema, suppressed neuronal degeneration, and improved neurological severity score, indicating that it may be involved in the secondary injury process of TBI. The link between CatS and secondary injury after TBI may be through regulation of the inflammatory process, as inhibiting it prevented the TBI-induced upregulation of proinflammatory factors in the brain cortex. Additional study of the roles of CatS in TBI might provide a new perspective of secondary brain injury after TBI and provide a novel target for therapy.

## Figures and Tables

**Table 1:** Neurological severity score (NSS).

Task	NSS	
	Failure/Success	
Exit a 30 cm diameter circle within 3 min	1	0
Absence of mono- or hemiparesis	1	0
Able to walk straight	1	0
Presence of startle reflex	1	0
Presence of seeking behavior	1	0
Able to walk on a 3 cm wide beam	1	0
Able to walk on a 2 cm wide beam	1	0
Able to walk on a 1 cm wide beam	1	0
Able to balance on a 1 cm wide beam for at least 10 sec	1	0
Able to balance on a round stick (0.5 cm wide) for at least 10 sec	1	0

Maximum total	10	

**Table 2:** Grip test scoring.

Task	Score
Unable to grasp wire for 30 sec	0
Grip wire for 60 sec with 1 or 2 paws	1
Jump up and grasp wire with 4 paws	2
Grasp wire with 4 paws and wrap tail around	3
Crawl along the wire for at least 5 cm	4
Crawl along wire to terminal and dismount	5

Maximum total	5
